# Relationship between the Grade and the Characteristic Flavor of PCT (Panyong Congou Black Tea)

**DOI:** 10.3390/foods11182815

**Published:** 2022-09-13

**Authors:** Chenxi Gao, Yan Huang, Jing Li, Shiheng Lyu, Zhihui Wang, Feng Xie, Yuqin Luo, Fan Zhang, Zhidan Chen, Weijiang Sun

**Affiliations:** 1 College of Horticulture, Fujian Agriculture and Forestry University, Fuzhou 350002, China; 2 Anxi College of Tea Science, Fujian Agriculture and Forestry University, Quanzhou 362400, China; 3 Fujian Xin Panyong Group Co., Ltd., Fuan 355000, China

**Keywords:** Congou black tea, chemical-physical, aroma, multivariate analysis, taste quantitative evaluation, quality grades

## Abstract

Panyong Congou black tea (PCT) is one of the most representative and historically famous Congou black teas in China and has been gaining more and more attention for its beneficial health properties. Currently, four grades of PCT are available, based on the raw leaf materials and consumer palatability. The chemical profiles distinguishing different grades of PCT are yet to be defined, nor has the relationship with grade been evaluated. In the present study, chemometric analysis showed that epigallocatechin (EGC), catechin (C), polyphenols, gallic acid (GA), and free amino acids are grade related bio-markers of PCT. These compounds are associated with the sweet and mellow aftertaste of PCT. A total of 34 volatile components were identified, of which the three component types with the highest relative percentages were alcohols (51.34–52.51%), ketones (27.31–30.28%), and aldehydes (12.70–13.18%). Additionally, our results revealed that sweet floral and fruity aromas were positively correlated with six volatile organic compounds (VOCs), 1-pentanol, propyl hexanoate, linalool, cyclohexanone, hexanal, and 2,5-dimethylpyrazine. Clear discrimination was achieved using orthogonal projections to latent structures discriminant analysis (OPLS-DA). The findings provide vital information on the characteristic flavor of each grade of PCT.

## 1. Introduction

**Figure 1:**
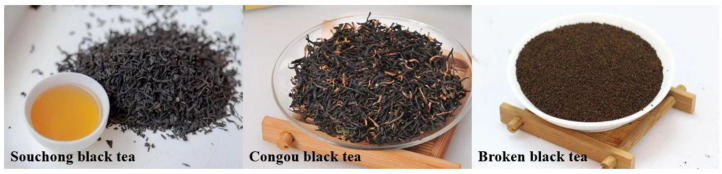
Different types of Chinese black tea.

As one of the most widely consumed teas, black tea accounts for approximately 75% of global tea consumption and is appreciated worldwide for its mellow taste, bright red color, distinctive flavor, and high nutritional value [1,2]. Black tea is planted in different provinces of China and has many varieties, but three main types have been identified: Souchong (Xiao Zhong) black tea, Congou (Gong fu) black tea, and Broken black tea (Figure 1) [3]. PCT is one of the most famous black teas in China. As a small-leaf type of Congou tea, it originates from Panyong Village in Fuan City. Raw materials of PCT are generally obtained from Panyong Caicha, Fu’an Dabaicha, or other locally planted tea varieties. PCT production generally includes a series of processes such as withering, rolling, fermentation, and drying [4]. PCT has a typical sweet and mellow taste due to adjustments in manufacturing technology and the elimination of the pine-smoke fragrance [5]. Currently, PCT is classified into four grades: super grade (SG), first grade (1G), second grade (2G), and third grade (3G) based on leaf morphology and sensory evaluation. SG and 1G are prepared from a few new, tender leaves from the top of the stem. 2G and 3G are composed of newly mature leaves. Although all grades of PCT are produced using the same standard procedure, their aroma and taste differ significantly. Currently, the discrimination of PCT quality mainly depends on basic chemical detection and sensory evaluation. Sensory evaluation, which is conducted in panel tests, is time consuming and laborious when a large number of samples must be assessed. Thus, the development of an objective, accurate, and rapid instrumental method for the discrimination of different quality grades of PCT is highly desirable to assist the panel test through a fast preclassification of samples and increase the efficiency of quality control in the market.

As is known to all, the comprehensive quality of a tea infusion is mainly reflected in three aspects: color, taste, and aroma [6]. A high-quality tea infusion always has a bright red color, a mellow taste, and a strong fragrance. Among these parameters, taste is one of the important indicators that affect the consumers’ preferences and choices. To date, studies on the taste of black tea have mainly focused on different processing techniques and different types of black tea. For example, Wang et al. [7] explored the evolution of nonvolatile compounds during the fermentation of Congou black tea, identified the precursors of key quality components, and found that withering promoted the conversion of catechins, yielding theaflavins (TFs) and thearubigins (TRs). Hua et al. [8] showed that Congou black tea has a sweet and mellow taste due to its extremely high catechins, amino acids, and tea pigment contents. Biochemical compounds such as tea polyphenols (TPs), amino acids, caffeine, TFs, TRs, and other large polymers are important substances in black tea extracts [7,9]. These components substantially alter the original compounds in raw tea leaves and confer unique sensory quality and health-promoting benefits to black tea (e.g., antioxidant, anti-inflammatory, anti-tumor, and metabolic regulatory effects) that might protect against metabolic, cardiovascular, and inflammatory diseases [10,11,12,13]. Furthermore, Congou black tea has been reported to protect against cancers of the esophagus, reduce the risk of a combination of alcohol drinking and smoking, and has pharmacological potential [14]. In addition to its nonvolatile compounds, the aroma of black tea is also crucial to the choice of consumers. VOCs are aroma components of tea that mainly include alcohols, aldehydes, and ketones. β-Ionone, benzeneacetaldehyde, geraniol, linalool, coumarin, β-damascone, and vanillin are signature aromas of special grade Keemun black tea [15]. Ma et al. [16] found that high contents of linalool, geraniol, and trans-β-ionone produced during black tea fermentation provide sweet flower and fruit aromas. Shi et al. [17] systematically showed that higher concentrations of heptanal, (Z)-4-heptenal, 2-hexenal, (E, E)-2,4-heptadienal, (E, E)-2,4-hexadienal, and (E)-2-octenal may be favorable to maintain the freshness of black tea, since they tend to provide a green and floral flavor at low levels.

Currently, gas chromatography-mass spectrometry (GC-MS) is a common analytical technique that is commonly used in aroma analyses, with the advantages of high qualitative and quantitative capabilities. However, limited by the detection threshold and multiple peak overlap, some compounds present at low concentrations may not be detected [18]. Headspace-gas chromatography-ion mobility spectroscopy (HS-GC-IMS) is a sensitive, state-of-the-art aroma detection instrument that has been used to detect volatile compounds with simple treatment. In previous studies, HS-GC-IMS has been widely applied in the food industry including flavor analyses of olive oil [19], ham [20], and honey [21] as well as determining wine origins [22]. Since 2020, an increasing number of studies have focused on the use of GC-IMS to analyze tea. The main studies are listed in Table 1. To our knowledge, HS-GC-IMS, chemical-physical, and quantitative descriptive analysis, (QDA) in combination with multivariate analysis for PCT grade determination, has not yet been reported.

We hypothesized that the four grades of PCT differed in their chemical profiles, which would potentially be distinguished by certain differentiated metabolites, and these profiles might be associated with sensory effects. Given the lack of existing information, the objectives of this study were to analyze and compare the chemical profiles among the four grades of PCT using chemical-physical, HS-GC-IMS, and QDA coupled to chemometrics methods to identify the grade related metabolites and to evaluate sensory effects using co-expression networks. Based on these data, we propose a new strategy for grading PCT by analyzing the combination of sensory effects and metabolic components.

## 2. Materials and Methods

### 2.1. Sample Preparation

The four different grades of PCT including SG, 1G, 2G, and 3G used in this study were procured from the Fujian Xin Panyong Group Co., Ltd. (Fuan, Fujian Province, China). SG and 1G were composed of a few newly tender leaves from the top of the stem. 2G and 3G were made of newly mature leaves. All of these samples were processed using traditional PCT processing techniques including withering, rolling, fermentation, and drying during the harvest season of May 2020. The different grades of PCT were obtained from the same refinement processing line for an accurate comparison. The PCT materials were sealed and stored at 5 °C in the refrigerator for the compound analysis and flavor evaluation.

### 2.2. Chemicals

All chemicals used for liquid chromatography in this study were of chromatographic grade. Acetonitrile, methanol, and formic acid were purchased from Merck (Darmstadt, Germany). Phenolic compounds, L-theanine, and caffeine standards were acquired from the Sigma–Aldrich Corporation (St. Louis, MO, USA) and Merck Chemicals (Merck, Darmstadt, Germany). n-Alkanes C9–C27 were purchased from the Sigma-Aldrich Corporation. The other chemical reagents were analytically pure and purchased from the MACKLIN Corporation (Shanghai, China).

### 2.3. QDA for the Sensory Evaluation

The sensory characteristics were inspected and evaluated by seven professional tea tasters (three men and four women, 24~38 years old) from the College of Horticulture of Fujian Agriculture and Forestry University. All panelists were well-trained and certified in tea-organoleptic evaluations by the Occupational Skill Identification Center of China. Each evaluation was conducted in a special sensory inspected room at a temperature of 25 ± 1 °C. Three grams of tea were measured using the uniform heap sampling method, and their appearances were examined. Subsequently, boiling water (150 mL) was added to each corresponding teacup, the leaves were soaked for 5 min, and then the liquor was immediately drained into a special tasting bowl for evaluation by the panelists. The indices of appearance, aroma, color, and taste of the brewed tea were assessed using the QDA method, and the results were recorded. The QDA method was based on the sensory evaluation procedure of the ISO standard of sensory analysis (ISO 11035 Sensory analysis—Identification and selection of descriptors for establishing a sensory profile by a multidimensional approach). The scoring scale ranged from 0~5 points, where 0 = not perceived, 1 = weak, 2 = rather weak, 3 = average, 4 = rather strong, and 5 = strong. We reduced the number of descriptors at this stage by initially classifying the samples according to the geometric mean *M*, which is the square root of the product of the frequency, *F*, and the relative intensity, *I*, of each descriptor: *M* = √(*F* × *I*), where ‘*F*’ is the number of times the descriptor is mentioned divided by the total number of times it is possible to mention that descriptor, which is reported as a percentage, and ‘*I*’ is the sum of the intensities given by the whole panel for a descriptor over the maximum possible intensity for this descriptor, expressed as a percentage. Each sample was evaluated three times.

### 2.4. HS-GC-IMS Analysis

The volatile organic compounds were detected using HS-GC-IMS (FlavorSpec, G.A.S., Dortmund, Germany). PCT (0.2 g) was transferred into a 20 mL headspace bottle. The headspace injection conditions were as follows: incubation at 80 °C for 15 min, the injection needle temperature was 80 °C, and the injection volume was 200 μL. GC conditions: the chromatographic column was FS-SE-54-CB-1 15 mL × 0.68 mm AD × 0.53 mm, the carrier gas was N_2_ (purity >99.99%), and the carrier gas flow rate was an initial flow rate of 2 mL/min that was maintained for 10 min, flow ramp up to 150 mL/min in 10 min, and maintained for 30 min. IMS conditions: the drift tube temperature was 45 °C, and the drift gas velocity was 150 mL/min. The retention index (RI) was calculated using n-alkanes C9–C27 as external references with VOCal software in the GC-IMS device. The volatile compounds were preliminarily identified based on a comparison of the RI and the drift time with the NIST library and IMS database retrieval software obtained from G.A.S. (Dortmund, Germany). All measurements were conducted in triplicate for individual samples.

### 2.5. Physicochemical Analysis

#### 2.5.1. Determination of Differences in Color

The PCT infusion was prepared according to the sensory evaluation method. The International Commission on Illumination (L*, a*, b*) system was used to quantify the color of tea powders and tea infusions. L* represents lightness, a* indicates red (+a*) and green (−a*), and b* indicates yellow (+b*) and blue (−b*) [9].

#### 2.5.2. Determination of pH

PCT infusions were prepared using the method described in the ‘Sensory evaluation’ section, and their pH values were determined when the filtrates were cooled to room temperature. The pH of the tea infusion was determined with a pH meter (Seven Excellence, Mettler Toledo, Shanghai).

#### 2.5.3. Determination of TPs and Free Amino Acids

TPs were determined by the Folin–Ciocalteu colorimetric assay (GB/T 8313-2018) with slight modification using GA as a standard [33]. Briefly, 0.20 g of black tea powder was mixed with 5 mL of 70% (*v/v*) methanol and ultrasonically extracted (SB-5200DT; Scientz, China) twice at room temperature for 15 min. After centrifugation (5000× *g* for 15 min) and pooling, the black tea extract was obtained. A total of 1.0 mL of black tea extract, water (blank control), and GA solutions (10 μg/mL, 20 μg/mL, 30 μg/mL, 40 μg/mL, and 50 μg/mL, which were used to construct the standard curve) were separately transferred to a 15 mL tube, 5.0 mL of Folin–Ciocalteu reagent (10%, *v/v*) were added, and the sample was vortexed for 30 s. After reacting for 5 min, 4.0 mL of a Na_2_CO_3_ solution (7.5%, *v/v*) was added, vortexed for 30 s, and incubated at room temperature for 60 min. The content of TPs was determined at 765 nm using a UV–Vis Spectrophotometer (Puxi General Instrument Co., Ltd., Beijing, China). Three replicates were used for each sample.

The free amino acid content was determined by the ninhydrin colorimetric method according to GB/T 8314-2013 [34]. Glutamic acid was used as a standard. Then, 1.0 ± 0.001 g of the ground tea sample was put into a 500 mL beaker, and then 300 mL of boiling water was added. After extracting for 20 min in a 100 °C water bath, the sample was filtered with 15 cm diameter quantitative filter paper (Tezhong Co., Ltd., Hangzhou, China) and fixed to volume in a 500 mL volumetric flask. After, 1.0 mL of the extracting solution was placed in a 25 mL colorimetric tube, then 0.5 mL pH 8.0 phosphate buffer and 0.5 mL 2% ninhydrin solution were added. After 15 min of being placed in a water bath in boiling water, we fixed the volume to 25 mL after cooling. The content of free amino acids was determined at 765 nm using a UV–Vis Spectrophotometer (Puxi General Instrument Co., Ltd., Beijing, China). Three biological replicates were performed for every sample.

#### 2.5.4. Determination of TRs and TBs

The tea pigment (TR and TB) contents were quantified through systematic analysis after extraction with an organic reagent (ethyl acetate, ethyl alcohol, and n-butyl alcohol) [34].

### 2.6. HPLC Analysis

The contents of catechins, caffeine, GA, and TFs were determined using high-performance liquid chromatography (HPLC, Waters 2695, MA, USA) [35]. The tea infusions were filtered through a 0.45-μm Millipore filter before injection. The HPLC conditions for the 2998 PDA detector (Waters, MA, USA) were as follows: chromatographic column: Phenomenex ODS C18 (100 × 4.6 mm, 3 µm); injection volume: 10 μL; column temperature: 35 °C; mobile phase A: 2% acetic acid, mobile phase B: acetonitrile, flow velocity: 1.0 mL/min; wavelength 280 nm; gradient elution: 0 min, 100% A; 25 min, 68% A; 5 min, 100% A.

### 2.7. Data Processing

All experimental data were calculated as the average of three replicate experiments and reported as the means ± standard deviations. SPSS (Version 21, SPSS Inc., Chicago, IL, USA) was used to analyze the significant differences among different treatments. The flavor wheel and radar charts were generated in Excel. LAV 2.2.1 software was used to process the HS-GC-IMS data, GC × IMS library search software was used to identify the volatile compounds, and LAV software was used to generate fingerprints. Principal component analysis (PCA) and orthogonal projections to latent structures discriminant analysis (OPLS-DA) were performed using SIMCA-P+ 14.1 software (Umetrics, Umeå, Sweden). Heatmaps were generated with TBtools software (Guangzhou, China).

## 3. Results and Discussion

### 3.1. Sensory Evaluation Analysis

**Figure 2:**
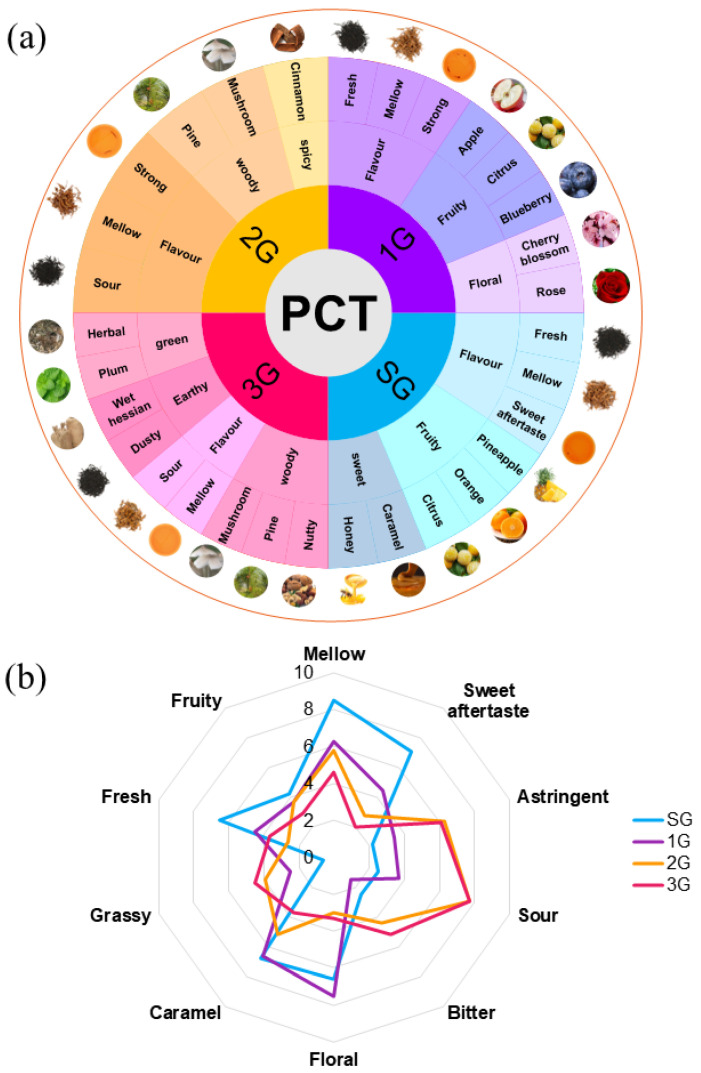
Sensory evaluation of the PCT standard samples. (**a**) Flavor wheel. (**b**) Sensory evaluation radar chart.

Flavor affects the acceptance and choice of food to consume. Based on the unique flavor and sensory impression, a flavor wheel of the flavor categories of PCT was developed from the descriptive analysis, as shown in Figure 2a. The system contains three types of descriptors: grade terms, first-tier terms, and second-tier terms. The design framework of the sensory wheel has a distinctive appearance, flavor lexicon, and sensory information and includes the infusion color and an image representing the smell. The first-tier contains familiar terms that represent flavors found in that grade. The second-tier terms name and define each separately identifiable flavor note in the PCT, and are used by the most expert and specially trained panels.

The traditional sensory evaluation of PCT showed that SG had a sweet aftertaste taste and released a pronounced sweet, fruity fragrance. The volatile flavor of the 1G sample was strong, with an outstanding floral flavor, while the taste was fresh and mellow. In 2G, sourness was the most important taste attribute, and the woody fragrance provided a spicy sensation. However, in the 3G group, the best volatile flavor described was strong grassy and earthy. The flavor characteristics in PCT were consistent with the order of the grade. This flavor wheel of PCT highlights the importance of aroma in the overall sensory character and provides more details on the reference standards and concentrations recommended for the training of these flavors in PCT, which facilitates the quality control of products [36,37].

After screening by the sensory group, five taste attributes and five odor attributes were selected for the descriptive analysis: “mellow”, “sweet aftertaste”, “astringent”, “sour”, “bitter”, “rose”, “caramel”, “grassy”, “fresh”, and “fruity”. The sensory intensity of the four products in different grades was quantitatively scored and shown in the PCT sensory vocabulary (Table 2). The quantitative description and analysis results of the flavor sensory evaluations of PCT were plotted in a radar chart, which is shown in Figure 2b. According to the assessors, the grade of SG exhibited the highest intensity of “mellow”, “sweet aftertaste”, and “fresh” flavor, but the lowest intensity of unpleasant notes such as “sour”, “astringent”, and “grassy” as well as moderate “caramel”, “floral”, and “bitter” odors. In addition, the taste of 1G PCT is moderate mellow and sweet, with the strongest floral aroma. Conversely, unpleasant notes such as sour, bitter, and astringent flavors were detected in the 2G and 3G groups.

### 3.2. Analysis of the Liquor Characteristics

Appearance plays a role in consumer acceptability, with color and clarity both identified as important factors that significantly influence the quality grades of PCT. Table 3 illustrates the visual appearance of tea infusions with different grades. Infusions brewed from 3G showed a much lighter color than those from SG. The visual observations of tea infusions were highly consistent with their chromatic parameters. The chromatic parameters of tea infusions brewed from the four grades were all significantly different (*p* < 0.05) (Table 3). The L* values for 2G and 3G were much higher than those for SG and 1G, indicating a lighter color. The a* values of the tea infusion decreased with decreasing grade level, indicating that the color of the high-grade PCT infusion was dark red. The values of b* decreased in the order of 3G > 2G > 1G > SG, indicating that the color of a low-grade PCT infusion was more yellow. Therefore, a visual discrimination of black tea from different grade levels could be achieved even based on color [38].

**Figure 3:**
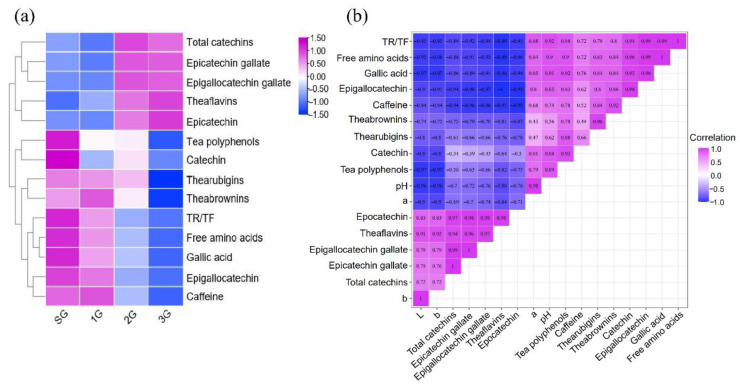
(**a**) Heatmap of the biochemical components in different grades of PCT. (**b**) Heat map based on the correlation analysis of compounds in PCT.

The pH of different grades in PCT samples varied based on (*p* < 0.05), ranging from 5.07 to 5.33. The better quality PCT sample exhibited a higher pH value; thus, the “sour” taste in the aforementioned sensory evaluation was strong in the 2G and 3G samples. pH is the crucial factor that influences the quality of tea extracts. The pH affects the activities of polyphenol oxidase and other enzymes, which also alters the oxidation of catechin and the formation of theaflavin to determine the quality of the tea (Figure 3b) [39,40].

Nonvolatile tastants and compounds also contribute to mouth-feel characteristics (e.g., amino acids, catechins, and polyphenols). It is the complexity of the interaction of these compounds in the matrix of tea products that determines the taste of PCT [41]. A heatmap was constructed and colored by the relative content change (Z score) after the normalization of the biochemical compositions in different PCT grades, and the results showed that SG and 1G contained greater free amino acids, TPs, EGC, C, TRs, TBs, caffeine, and GA contents and higher TF/TR values. In contrast, SG and 1G had lower contents of TFs, EC, EGCG (epigallocatechingallate), ECG (epivcatechingallate), and total catechins (Figure 3a).

Amino acids and catechins are important hallmarks of black tea, and TFs, TRs, and TBs are critical substrates for the formation of quality-related components [7,42]. According to Figure 3a, the highest total amount of free amino acids was present in SG (3.3%), followed by 1G (2.99%) > 2G (2.45%) > 3G (2.15%). The levels of amino acids exert a significant sensory effect on fresh and sweet taste of tea products. Yu et al. [43] also reported that the free amino acid content was positively correlated with the tea grade, consistent with our results.

TPs exert various effects on health including anticancer activity and preventive effects on diabetes and cardiovascular diseases [44], and they are important components that affect the formation of a sweet aftertaste, thick, strong, refreshing, and bitter tastes of tea infusions [43]. Figure 3a shows that SG contained the highest TP content (13.58%), which was approximately twice that in 3G (8.06%). Thus, maintaining a certain proportion of TPs might ensure a high level of fresh and refreshing taste of a high-grade black tea after long-term fermentation. Hua et al. reported that fermentation favored the maintenance of polyphenol oxidase activity and the continuous formation of TFs, theasinensins, and TRSI (a TR fraction), resulting in better *L* and *b* values and liquor color [45]. Therefore, they might be potential grade-related compounds.

Catechins account for approximately 70% of TPs and contribute the astringent taste to the tea infusion. In particular, the composition and concentration of EC constitute not only the main body of the astringency, but also the thickness of the tea infusion and tea quality. Among the catechin components detected in this study, the contents of EC, ECG, ECCG, and total catechins in low grades (2G and 3G) were significantly higher than those in SG and 1G, which might explain the astringent taste of low-quality PCT. However, the contents of C and EGC in SG and 1G were significantly higher than those in 2G and 3G. In previous studies, long-term fermentation was shown to significantly decrease the total amount of catechins [7]. In our study, it was also found that the total catechin content in high-grade black tea was significantly lower than that in low-quality tea (2G and 3G). Therefore, high-grade PCT is usually deep-fermented [46]. Qin et al. found that the hydrolysis of galloylated ECG and EGCG in high-grade black tea can lead to an increase in nongalloylated C and EGC contents, giving PCT a sweet aftertaste taste [47]. This finding might explain why catechin and EGC concentrations progressively decreased from higher to lower grades of PCT.

Tea pigments including TFs, TRs, and TBs contribute to the color of the black tea infusion [48]. The concentrations of tea pigments (TFs, TRs, and TBs) in the tea infusions are shown in Figure 3a and Table S1 (Supplementary Materials). A lower level of TFs was detected in tea infusions of samples SG and 1G, and higher concentrations of both TRs and TBs produced a redder infusion (Table 2). The tea infusions of samples 2G and 3G contained higher concentrations of TFs but lower concentrations of TRs and TBs, which produce a lighter color in infusions. Previous studies reported that the content of theaflavin in the tea infusion was significantly correlated with *L* * and *b* *, consistent with our study (Figure 3b) [9,49]. In fact, the fresh leaves used to prepare low-grade black tea are always mature or old, and insufficient fermentation during processing leads to a higher proportion of theaflavin, resulting in a yellow tea liquid with a grassy and astringent taste. A higher *a* * value represents a redder color of the black tea infusion and a higher content of TRs in black tea, which results from the efficient fermentation of black tea [50]. The ratio of TR to TF is an important index for evaluating the black tea quality [51]. In our study, TR/TF values decreased with decreasing grade. Bhuyan et al. reported that when the ratio of TR/TF was high, the infusion color was bright, and a low infusion concentration may result in a negative effect on the taste [52]. Thus, the analysis of tea pigments showed that pigments in the tea infusion were positively related to the color of the PCT infusion.

GA and caffeine are responsible for the sweet aftertaste and bitterness of black tea. The difference grade resulted in significant differences in GA and caffeine contents (*p* < 0.05) (Figure 3). The content of GA in SG (1.88 mg/g) was comparable to that in 1G (1.75 mg/g) and higher than that in 2G (1.58 mg/g) and 3G (1.46 mg/g). Zhou et al. [53] reported that the GA content in tender leaves was higher than that in the old leaves. Furthermore, EGCG was oxidized, consumed, and hydrolyzed to form C, EGC, and GA during fermentation, consistent with the conclusion that high-grade PCT contains higher C, EGC, and GA concentrations. Considering the tenderness of raw materials for each grade of PCT, these results well-support our finding that GA levels gradually decreased from higher grades to lower grades. Although the difference was not significant, the higher caffeine content in high-grade PCT may be due to the higher tenderness of the raw material, which also adds a rich taste to the extract.

### 3.3. VOC Analysis

#### 3.3.1. GC-IMS Topographic Plots of Different Grades of PCT

**Figure 4:**
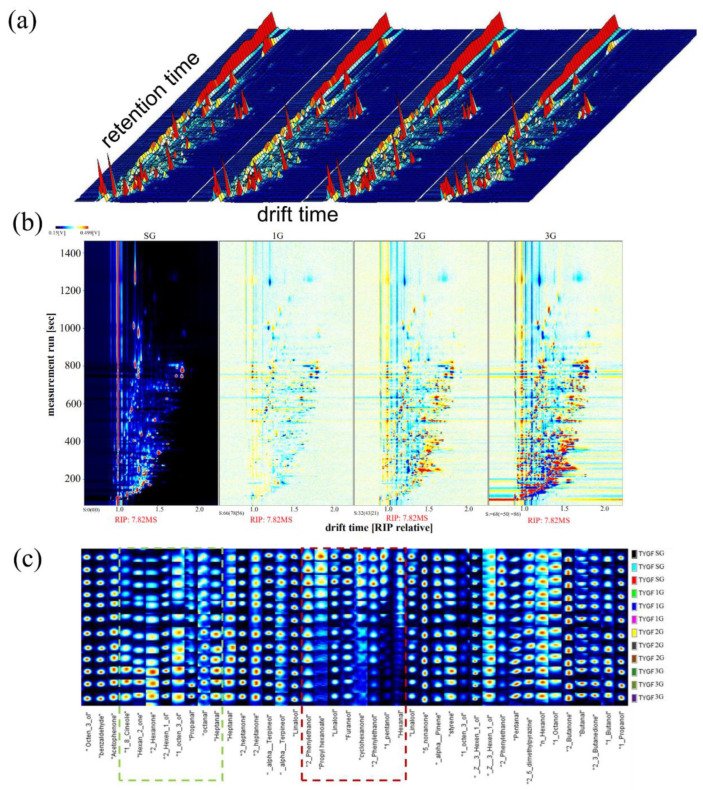
A comparison of the HS-GC-IMS spectra of the VOCs. (**a**) The 3D topographical plots for VOCs with peak spectra. The vertical coordinate represents the retention time of gas chromatography, whereas the horizontal coordinate represents the ion migration time. The background of the whole figure is blue, and the red vertical line at abscissa 1.0 is the RIP peak (reactive ion peak, after normalization). (**b**) The two-dimensional graph with the super-grade as a reference. (**c**) Gallery plot of the selected signal peak areas obtained for different grades of the PCT standard samples. As the grade decreases, the red outline indicates VOCs with decreased intensities, and the green outline indicates VOCs with increased intensities.

The HS-GC-IMS method was applied to obtain global IMS information from the samples, with the aim of identifying the volatile components and the regularity of the variation in the different grades of PCT. The volatile intensity signals in SG, 1G, 2G, and 3G analyzed by HS–GC–IMS were visualized in a 3D topographic plot (Figure 4a).

For the convenience of observation, the top view of the differential contrast model was used to compare differences in the compounds between samples. Each point on both sides of the RIP peak indicates a flavor substance. The SG sample was used as a reference, and all of the samples were subtracted from the reference. If the intensity of VOCs was close, the background of the topographic map deduced from the other samples is shown as white spots, red means that the concentration of the substance is higher than the reference, and blue indicates that the substance concentration was lower than that in the reference substance. In the differential contrast model plot (Figure 4b), as the grade changes, many red and blue spots have been observed, indicating that many VOCs were different from those in the control group. As shown in Figure 4b, most of the signals were located in the retention time range of 100–300 s, and the VOCs changed inconspicuously in a drift time range of 1.0–1.8 in the topographic plot. Compared with the reference, more blue and red spots at drift times of 1.5–1.8 were observed in the plots of different samples, showing that the increase or decrease in the levels of these substances led to aroma variations in different grades of PCT. Thus, the GC-IMS spectra were clear and easily characteristic of the volatile profiles of different grades of PCT. The comprehensive volatile profile information of different samples proved necessary for the in-depth statistical analysis (see Table 4).

The characteristic fingerprint was established to clearly observe the differences in the signals for VOCs and search for suitable markers in different grades of PCT, as shown in Figure 4c. The signal intensity of the volatile compounds in the plot of SG, 1G, 2G, and 3G were confirmed, and the characteristic VOCs of different grades of PCT were also labeled. In the area labeled with a red rectangle, the signal intensities of 2-phenylethanol, propyl hexanoate, linalool, furaneol, cyclohexanone, 1-pentanol, and hexanal became weaker with decreasing PCT grade, which was advantageous to the formation of the typical smell of high-grade black tea. In areas labeled with the green rectangle, the 1,8-cineole, hexan-2-one, 2-hexen-1-ol, 1-octen-3-ol, propanal, octanal, and heptanal contents increased and showed stronger signal intensities with decreasing PCT grade, which caused the unpleasant taste of low-grade black tea. According to the fingerprints, the volatile profiles of different grades of PCT and characteristic VOCs responsible for differences between grades were easier to identify. A similar result was also reported in a study of the flavor fingerprint of Oolong tea of different origins [30].

#### 3.3.2. Analysis of VOCs in Different Grades of PCT

High-quality PCT often has a pleasant flavor, and the substances that produce flavors were significantly and positively correlated with quality and popularity. The VOCs in PCT were characterized and qualitatively analyzed by comparing the IMS retention index and drift time with the control. The results are shown in Table 4 and Table S2. After analyzing the standard substances and RI similarities, a total of 42 compounds were identified in the PCT samples including 15 alcohols, 10 ketones, nine aldehydes, three terpenes, one nitrogen heterocyclic compound, and one ester. The identified VOCs are represented by Nos. 1~34 in Table 4 and are probably derived from the Maillard reaction or the thermal degradation of lipids [54]. However, some VOCs show high proton affinity, and monomers form dimers or even trimers during the migration process [55]. The VOCs were quantified using the normalization method based on their peak volumes calculated in the IMS system.

Alcohols are important major aroma compounds in black tea that are products of the Maillard reaction and present floral, fruity, and grassy odors [54]. The relative content of alcohols was the highest among the VOCs, ranging from 51.34% to 52.51% in different grades of PCT samples (Figure S1). It was reported that alcohols identified in black teas ranged from 28.33% to 58.33% of the total volatiles present [56,57], which was similar to our results. Among these alcohols, linalool (caramel), 2-phenylethanol (rose), and 1-pentanol (rose) showed the highest signal intensities in SG, which were reported as the key aroma compounds of superior black tea [58]. These VOCs can be chosen as good quality indicators of PCT. Conversely, 1,8-cineole (herbal), 1-octen-3-ol (mushroom), (Z)-3-hexen-1-ol (grassy), and 2-hexen-1-ol (grassy) are alcohols whose signal intensity increases with decreasing grade and have also been detected in black tea by He [59] and Su [15].

Ketones are another group of important VOCs that are generally present in black tea [60] and are generally the products of β-carotene oxidative degradation [61]. A total of nine ketones were identified in black tea samples, accounting for 27.31–30.28% of the total VOCs. The composition of the main ketones in the PCT extract was dominated by 5-nonanone and 2-butanone, which exhibit rich and complex aromas of fruit and green color [62]. In SG, furaneol and cyclohexanone showed the highest intensity and produced caramel and minty aromas. In addition, the signal intensity of hexan-2-one (mushroom) was negatively correlated with the PCT grade.

The nine aldehydes identified in the PCT samples accounted for 12.70–13.18% of the total VOCs and are produced by the oxidative degradation of amino acids (mostly phenylalanine) and lipid oxidation in tea [63]. The main aldehydes in PCT are hexanal and benzaldehyde, which provide grass and floral aromas to high-grade samples. Hexanal is mainly derived from the oxidation of linoleic acid during deep fermentation. From SG to 3G, the signal intensity of hexanal gradually decreased, indicating that the aroma of roses was also lower in low-grade samples [56]. In contrast, the signal intensity of octanal, heptanal, and propanal increased with decreasing grade, indicating more citrus and grass flavors in low-grade PCT.

A total of three types of terpenes were identified in the PCT samples, accounting for 4.55–5.65% of the total VOCs. It has been reported that terpenes are one of the most important compounds affecting the formation of the tea aroma [64]. As shown in Table 3, the contents of limonene (minty) and styrene (fruity) in the low-grade PCT were higher than those in the other samples. α-Pinene showed the highest signal intensity in SG, which releases a sweet fruity aroma such as blueberries [17].

The contents of nitrogen heterocyclic compounds (2,5-dimethylpyrazine) and esters (propyl hexanoate) were 0.82–1.01% and 0.13–0.36%, respectively. The signal intensity of the nitrogen heterocyclic compounds decreased with decreasing grade, imparted the flavor of roasted aroma [65], and had a high aroma intensity and low aroma threshold [66]. Esters mostly present fruity and fresh aromas, which play significant roles in the formation of the black tea aroma. Ester components arise from the dehydration condensation of higher fatty acids and lower alcohols [67].

In general, in the different grades, the composition of VOCs in tea infusion varieties results in different tea aromas. However, in our study, no significant difference in the total amount of various aroma components were observed between different grades of PCT. The high concentration of a VOC does not exactly mean that it is an important contributor to aroma (Figure S1). The differences in key aroma active compounds should be considered in the establishment of aroma types for different grades of PCT.

#### 3.3.3. Key Aroma-Active Compounds of Four Grades PCT

**Figure 5:**
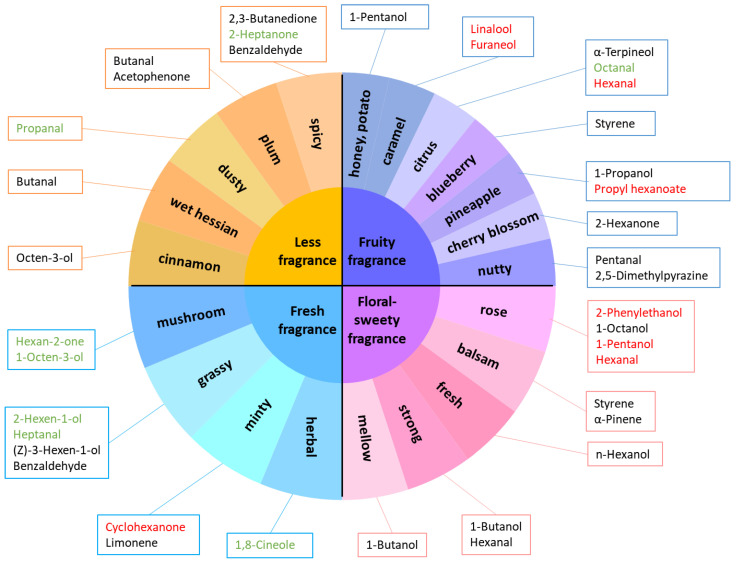
Key VOCs forming the four characteristic aroma types. Compounds marked in red indicate that the highest content was at high grade PCT, and those marked in green indicate that the highest content was at low grade PCT.

According to the contents of VOCs in PCT, four aroma types were established: (1) fruity fragrance; (2) floral-sweety fragrance; (3) fresh fragrance; and (4) less fragrance (Figure 5). PCT belongs to the first category in terms of the contents of the main VOCs. Furthermore, based on the aroma characteristics of volatile components with high content, the aroma components with fruity and floral aromas are considered the characteristic aroma compounds of high-grade PCT [68]. The components that produce a strong odor of caramel, citrus, and pineapple include linalool, furaneol, hexanal, and propyl hexanoate; and some compounds provide a rose aroma including 2-phenylethanol and 1-pentaol [69]. On the other hand, components with grassy aromas are considered as the characteristic aroma compounds of low-grade PCT. The following components are VOCs with grassy and herbal aromas: 2-hexen-1-ol, heptanal, and 1,8-cineole. Some compounds have a mushroom aroma including hexan-2-one and 1-octen-3-ol, and other compounds have dusty and spicy aromas including propanal and 2-heptanone [54]. These VOCs were present at much higher contents in 2G and 3G PCT.

Although C6–C9 alcohols and aldehydes generally impart strong grassy odors, when they are converted into corresponding esters, they generally produce pleasant fruity, floral, or fresh odors [70,71]. In black tea, adequate fermentation may reduce the content of alcohols including (Z)-3-hexen-1-ol, 1-hexanol, and 1-octen-3-ol and the levels of aldehydes such as heptanal and propanal. Sweety is the typical aroma for black tea, while floral and fruity aromas make black tea more attractive and have a better aroma quality. Therefore, the pleasant aroma of high-grade black tea is derived from deep fermentation, which is also reflected in the biochemical components.

### 3.4. Multivariate Statistical Analysis of Biochemical Compositions and VOCs

Chemometrics is a common multivariate classification method for grouping similar samples [72]. Chemometrics such as PCA and OPLS-DA have been used to discriminate samples and classify and verify data [34,73].

#### 3.4.1. PCA of the Four Different Grades of PCT

**Figure 6:**
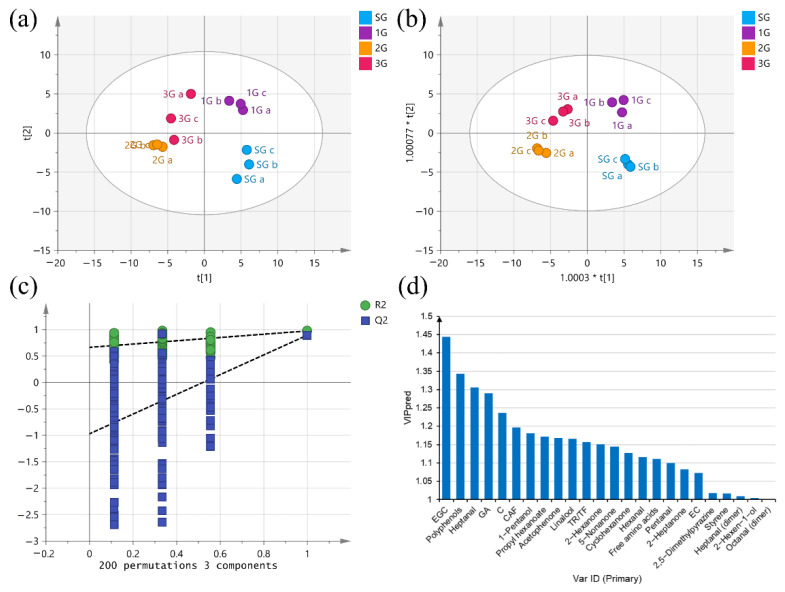
The PCA score plot and OPLS-DA score plot of PCT. (**a**) PCA score chart, R^2^X [1] = 0.495, R^2^X [2] = 0.208, Q^2^ = 0.566; (**b**) OPLS-DA score chart, R^2^X [1] = 0.491, R^2^X [2] = 0.189, Q^2^ = 0.781. (**c**) Cross-validation plot of the OPLS-DA model, R^2^ = 0.664, Q^2^ = –0.971. (**d**) VIP plot of markers with VIP > 1.

Fourteen biochemical compositions and 34 VOCs from four different grades of PCT samples were subjected to PCA to visualize the similarity of the samples (Figure 6a). The first and second principal components explained 49.5% and 20.8% of the total variance, respectively. Four PCT samples were gathered in the middle of the PCA score plot, and the three replicates of each sample were effectively aggregated, indicating good repeatability and reliable data. Intriguingly, the 2G and 3G samples were closely clustered. A clear separation of the 2G/3G samples from the SG and 1G samples was observed, while the SG and 1G samples were also clearly separated from each other.

#### 3.4.2. OPLS-DA of the Four Different Grades of PCT

Compared with PCA, OPLS-DA provides a supervised classification that effectively distinguishes samples and extracts the information for different variables [74]. OPLS-DA was applied to maximize the separation of samples and better distinguish the main differences between samples of different grades. The OPLS-DA score plot showed that four different grades of PCT samples were clearly separated (Figure 6b). The model showed a high degree to explain the differences between samples (R^2^X [1] = 0.491, R^2^X [2] = 0.189, Q^2^ = 0.781). The permutation test was repeated 200 times and indicated that the model was not overfitted (R^2^ = 0.664, Q^2^ = −0.971) (Figure 6c). As shown in Figure 6d, according to the principle of a VIP value over 1.0, a total of 23 compounds and TF/TR indicators were screened that were closely related to the grades of PCT including free amino acids, polyphenols, catechins (EGC, C and EC), GA, CAF, aldehydes (heptanal, hexanal, pentanal, heptanal (dimer) and octanal (dimer)), ketones (acetophenone, 2-hexanone, 5-nonanone, cyclohexanone, and 2-heptanone), alcohols (linalool, 2-hexen-1-ol, and 1-pentanol), terpenes (styrene), esters (propyl hexanoate), and nitrogen heterocyclic compounds (2,5-dimethylpyrazine). All the VIP values are ranked in Table S3.

### 3.5. Relationship between Biochemical Compositions, VOCs and Sensory Characteristics of PCT

**Figure 7:**
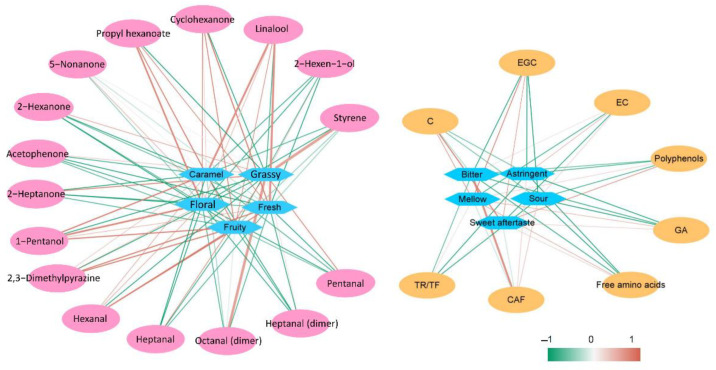
The correlation network between different VOCs, biochemical compositions, and sensory characteristics of different grades of PCT. The diamond represents the taste and aroma, and the ellipse represents the VOCs and biochemical compositions. The red line indicates a positive correlation, the green line indicates a negative correlation, and a darker color indicates a stronger correlation.

The biochemical compositions and VOCs can be used to accurately identify the grade of black tea [72,73,74,75]. The correlation coefficient and correlation network diagram between compounds with VIP > 1 and flavor are shown in Table S4 and Figure 7. Among the seven biochemical compositions, EGC, C, polyphenols, GA, and free amino acids were positively correlated with “sweet aftertaste” and “mellow” intensity, and caffeine and EC were positively correlated with “sour” and “bitter” intensity (Figure 7, Table S5). In the sensory evolution, the “sweet aftertaste” and “mellow” intensity in SG and 1G was stronger than that in 2G and 3G. The sweet aftertaste of tea is often detected after bitterness and astringency, and it is always used as a positive term to describe tea infusions [76]. Polyphenols, catechins, GA, and amino acids are strongly associated with attributes of the sweet aftertaste of PCT [77,78,79]. However, the high levels of caffeine result in bitterness and negatively affect taste; therefore, its decreased accumulation contributes to an improved quality of low-grade PCT [80]. This finding may be due to the thin taste of low-grade PCT, which makes it easy to highlight bitterness.

Among the 16 VOCs, 1-pentanol, propyl hexanoate, linalool, cyclohexanone, hexanal and 2,5-dimethylpyrazine were positively correlated with “fruity”, “floral”, “caramel”, and “fresh” (Figure 7). In the aroma classification of VOCs (Figure 4), 1-pentanol, propyl hexanoate, linalool, cyclohexanone, hexanal, and 2,5-dimethylpyrazine were present at the highest contents in high-grade PCT, which produced fruity, caramel, and flower flavors. In addition, heptanal, acetophenone, and 2-heptanone were positively correlated with “grassy” flavors. These VOCs were present at the highest contents in low-grade PCT and usually produce an unpleasant smell, which indicates low-quality black tea. Therefore, the differences in the quality of different grades of PCT are mainly derived from biochemical compositions such as free amino acids, polyphenols, catechins, GA, and CAF and VOCs such as aldehydes, ketones, alcohols, terpenes, esters, and heterocyclic nitrogen compounds.

## 4. Conclusions

PCT is one of the most popular and famous traditional full-leaf black teas in the world, but its chemical quality across different grades has remained largely unexplored. The present study aimed to explore the relationship between the grade and the characteristic flavor of PCT. Chemical-physical analysis, HS-GC-IMS, and QDA in combination with a multivariate analysis were applied to analyze four different grades of PCT. The results revealed 14 biochemical components and 34 VOCs, which can be used to identify different grades of PCT. The 2G and 3G PCT samples had similar VOC and biochemical compositions but differed from the SG and 1G PCT, and the OPLS-DA model showed a good ability to explain the sample variation (R^2^X [1] = 0.491, R^2^X [2] = 0.189, Q^2^ = 0.781). Furthermore, the OPLS-DA results indicated that EGC, C, polyphenols, GA, and free amino acids are factors with a positive effect on the sweet aftertaste and mellow taste of high-grade PCT, and 1-pentanol, propyl hexanoate, linalool, cyclohexanone, hexanal, and 2,5-dimethylpyrazine positively affected the sweet floral and fruity aromas. In summary, these results provide a new strategy to distinguish tea grades and can be applied not only to tea, but also to other agricultural products.

The results of this study provide a preliminary grading strategy for different grades based on the existing PCT samples. With the enrichment of PCT categories, our findings will need to be verified and supplemented in the future to ensure the richness and comprehensiveness of PCT samples. In addition, mineral elements, antioxidant capacity, and other factors represent different aspects of tea quality. In the future, a more comprehensive database of black tea will be established, and big data will be used to predict unknown samples, origin, grade, year, and other quality factors.

## Figures and Tables

**Table 1:** The main results from studies using GC-IMS to analyze tea that have been published since 2020.

Tea Sample	Research Goal	Pattern Recognition Method	Reference
Chinese green tea	Quality change in manufacturing process analysis	PLS-DA/OPLS-DA	[23]
Liupao tea		PCA	[24]
Chinese citrus tea	Tea biochemical content analysis	OPLS-DA	[25]
Yingde black teas		PLSR/PCA	[26]
Chinese white tea		PCA/LDA	[27]
Chinese green tea		PCA	[28]
Chinese oolong tea	Tea origin identification	PCA/OPLS-DA	[29,30]
Chinese white tea		LDA-KNN/MLP/SVM/Adaboost/DecisionTree/KNN/MLP/Random Forest/SVM	[31]
Rwanda black/green tea	Tea quality analysis	PCA	[32]

**Table 2:** The geometric mean (*M*) values of the sensory attributes of PCT.

Types	Attributes	*F*	*I*	*M*
Taste	Mellow	0.857	0.493	0.65
	Sour	0.750	0.414	0.56
	Astringent	0.714	0.307	0.47
	Sweet aftertaste	0.607	0.300	0.43
	Bitter	0.571	0.214	0.35
Aroma	Floral	0.679	0.429	0.54
	Caramel	0.786	0.429	0.58
	Fresh	0.571	0.357	0.45
	Fruity	0.429	0.300	0.36
	Grassy	0.464	0.214	0.31

**Table 3:** The L*, a*, and b* values and pH of different grades of PCT infusions.

Grade	*L*	*a*	*b*	pH	Picture
SG	13.14 ± 0.36 d	17.78 ± 0.50 a	22.79 ± 0.56 c	5.33 ± 0.02 a	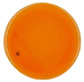
1G	15.50 ± 0.06 c	15.46 ± 0.12 b	26.73 ± 1.82 b	5.15 ± 0.01 b	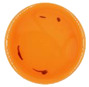
2G	16.27 ± 0.37 b	14.51 ± 1.11 b	28.05 ± 0.63 b	5.09 ± 0.04 c	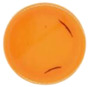
3G	17.70 ± 0.72 a	14.89 ± 1.25 b	30.51 ± 1.23 a	5.07 ± 0.01 c	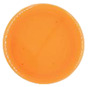

Note: *L*—brightness; *a* and *b*—chromaticity coordinates; the value of chromaticity A corresponds to changes from red (+*a*) to green (−*a*), and the value of chromaticity B corresponds to changes from yellow (+*b*) to blue (−*b*). a–d Different letters indicate significant differences among the four grades of PCT (*p* < 0.05).

**Table 4:** The identification of VOCs in four grades of PCT based on GC-IMS.

Code	Classification	Aroma Constituent ^a^	Aroma Description ^b^	Intensity (V) ^c^			
				SG	1G	2G	3G
1	Alcohols	Octen-3-ol	Mushroom	3886.33 ± 100.66 b	3609.45 ± 134.40 c	3943.22 ± 54.50 b	4177.67 ± 71.03 a
2		α-Terpineol^M^	Citrus, lemon	2949.57 ± 63.95 a	2577.49 ± 104.25 c	2809.92 ± 23.77 b	2568.83 ± 63.38 c
2′		α-Terpineol^D^	Citrus, lemon	524.75 ± 22.96 b	520.25 ± 5.04 b	536.04 ± 9.65 ab	561.62 ± 16.45 a
3		Linalool^M^	Caramel, orange	4647.72 ± 15.23 a	4461.71 ± 33.46 b	4374.99 ± 37.82 bc	4293.98 ± 77.49 c
3′		Linalool^D^	Caramel, orange	1427.40 ± 5.69 a	969.31 ± 2.87 b	847.85 ± 6.77 c	816.02 ± 7.24 d
4		1,8-Cineole	Herbal, medicinal	238.33 ± 2.53 d	253.55 ± 4.08 c	387.17 ± 5.03 b	516.52 ± 16.55 a
5		(Z)-3-Hexen-1-ol^M^	Grassy, fresh	5505.77 ± 68.70 b	5644.36 ± 181.58 b	6562.79 ± 3.75 a	6654.21 ± 47.38 a
5′		(Z)-3-Hexen-1-ol^D^	Grassy, fresh	211.42 ± 13.19 b	240.90 ± 32.70 ab	264.57 ± 11.46 a	253.79 ± 9.59 a
6		1-Octen-3-ol^M^	Mushroom, green	735.75 ± 8.31 d	806.05 ± 7.50 c	949.14 ± 15.96 b	1247.91 ± 10.67 a
6′		1-Octen-3-ol^D^	Mushroom, green	217.70 ± 12.13 a	149.86 ± 42.96 b	246.01 ± 6.60 a	225.12 ± 31.38 a
7		2-Hexen-1-ol	Grassy, fruity	775.19 ± 9.62 d	836.73 ± 5.66 c	935.48 ± 4.89 b	952.85 ± 5.48 a
8		2-Phenylethanol^M^	Rose	1367.68 ±29.44 a	1121.66 ±13.75 b	1111.16 ±2.40 c	834.50 ±2.81 d
8′		2-Phenylethanol^D^	Rose	624.30 ± 22.11 a	399.38 ± 113.11 b	397.28 ± 14.66 b	232.15 ± 9.80 c
9		n-Hexanol	Fresh, fruity	282.98 ± 12.54 b	336.49 ± 47.18 a	321.37 ± 12.10 ab	363.71 ± 8.95 a
10		1-Octanol	Rose, sweet	413.14 ± 28.80 a	353.58 ± 42.80 b	385.25 ± 6.27 ab	327.58 ± 6.00 c
11		1-Pentanol	Honey, potato	1192.90 ± 206.94 a	578.49 ± 244.22 b	267.42 ± 2.90 c	221.55 ± 2.30 d
12		1-Butanol	Mellow, sweet	738.90 ± 7.34 c	906.53 ± 107.02 b	1014.82 ± 18.69 ab	1113.69 ± 89.36 a
13		1-Propanol	Pineapple, sweet	5549.51 ± 135.63 c	5830.68 ± 138.77 b	5896.31 ± 51.29 b	6173.71 ± 165.10 a
14	Esters	Propyl hexanoate	Pineapple, fruity	216.74 ± 15.59 a	141.15 ± 23.10 b	102.76 ± 5.56 c	78.65 ± 3.40 d
15	Ketones	5-Nonanone	Fruit, green	7112.43 ± 98.23 ab	6828.97 ±191.28 c	7308.86 ±76.61 a	6904.97 ±44.99 bc
16		Furaneol	Caramel	814.05 ± 5.10 a	670.88 ± 3.45 b	625.6 ± 58.65 c	626.8 ± 43.16 c
17		Acetophenone	Plum, bitter	662.51 ± 4.06 c	742.55 ± 21.35 b	874.06 ± 21.59 a	848.66 ± 38.97 a
18		2-Heptanone^M^	Spicy, coconut	1152.87 ± 63.64 b	1110.23 ± 94.47 b	1472.83 ± 17.64 a	1584.87 ± 43.77 a
18′		2-Heptanone^D^	Spicy, coconut	433.79 ± 2.85 a	373.8 ± 41.34 bc	416.17 ± 27.81 ab	360.18 ± 8.59 c
19		Cyclohexanone	Minty	317.31 ± 1.27 a	276.78 ± 7.60 b	174.41 ± 4.86 c	126.37 ± 8.68 d
20		Hexan-2-one	Mushroom, buttery	140.95 ± 6.55 c	145.19 ± 2.05 bc	160.99 ± 9.34 b	225.11 ± 15.50 a
21		2-Hexanone	Cherry blossom	80.14 ± 15.90 c	122.93 ± 23.19 b	149.19 ± 6.93 b	219.61 ± 6.94 a
22		2-Butanone	Minty	4696.86 ± 435.80 c	5363.33 ± 404.29 b	5341.69 ± 163.86 b	6080.01 ± 220.30 a
23		2,3-Butanedione	Spicy, buttery	1129.26 ± 61.13 b	1245.79 ± 75.49 a	1132.63 ± 18.72 b	1283.83 ± 54.23 a
24	Aldehydes	Benzaldehyde^M^	Grassy, wood	2507.51 ± 68.05 b	2501.47 ± 182.02 b	2526.64 ± 24.40 b	2845.44 ± 61.76 a
24′		Benzaldehyde^D^	Grassy, wood	855.95 ± 30.38 c	925.99 ± 34.53 bc	981.39 ± 18.48 b	1029.80 ± 17.62 a
25		Octanal	Citrus, orange	169.13 ± 3.56 c	170.82 ± 20.86 c	180.81 ± 10.42 b	215.92 ± 20.75 a
26		Heptanal^M^	Grassy, cilantro	232.63 ± 2.70 b	236.25 ± 6.63 b	272.70 ± 18.78 a	288.07 ± 14.16 a
26′		Heptanal^D^	Grassy, cilantro	100.64 ± 22.75 b	136.21 ± 24.11 b	202.07 ± 7.63 a	215.42 ± 27.27 a
27		Pentanal	Nutty	531.21 ± 7.91 d	583.68 ± 8.79 c	833.20 ± 21.02 a	753.50 ± 19.92 b
28		Butanal	Plum, malty	908.45 ± 29.88 a	786.44 ± 33.63 ab	915.30 ± 15.48 a	749.17 ± 47.17 b
29		Propanal	Dusty, earthy	1177.58 ± 2.28c	1190.51 ± 1.55b	1195.49 ± 4.96b	1205.05 ± 10.01a
30		Hexanal	Citrus, lingering	1496.42 ± 19.55 a	1302.1 ± 15.08 b	558.97 ± 28.67 c	407.69 ± 15.70 c
31	Terpenes	Limonene	Minty, fresh	340.14 ± 19.09 c	322.21 ± 31.03 c	389.95 ± 29.80 a	368.37 ± 17.91 ab
32		α-Pinene	Blueberry, herbal	2335.85 ± 68.76 a	2072.58 ± 457.49 ab	2235.77 ± 61.75 a	1653.22 ± 148.25 b
33		Styrene	Blueberry, floral	660.90 ± 13.90 ab	580.98 ± 74.08 b	706.45 ± 18.98 a	723.42 ± 62.80 a
34	Nitrogen heterocyclic compound	2,5-Dimethylpyrazine	Nutty	604.24 ± 30.34 a	591.33 ± 18.45 a	514.18 ± 18.26 b	493.06 ± 29.52 b

Note: ‘a’, VOC identified using the GC-IMS analysis based on a comparison with the RI and the mass spectra of standard compounds. M—monomer. D—dimer; ‘b’, The aroma characteristics of compounds were obtained with reference to http://www.thegoodscentscompany.com/index.html (accessed on 9 August 2021); ‘c’, Different letters in the same row indicate significant differences (n = 3, *p* < 0.05).

## Data Availability

The data presented in this study are available on request from the corresponding author.

## Supplementary Materials

The following are available online at https://www.mdpi.com/article/10.3390/foods11182815/s1. The data presented in this study are available within the manuscript and the Supplementary Materials. Figure S1: Aroma compositions in four grades of PCT obtained from the GC–IMS analysis; Table S1: Contents of the biochemical components in standard samples of different grades of PCT; Table S2: GC–IMS integration parameters of volatile compounds to distinguish different grades of PCT; Table S3: The VIP value; Table S4: Pearson correlation coefficient between the aroma and VOCs; Table S5: Pearson correlation coefficient between taste and biochemical composition.
