# Supplementary material for: Relationship between the Grade and the Characteristic Flavor of PCT (Panyong Congou Black Tea)
Source: Foods. 2022 Sep 13;11(18):2815. doi: 10.3390/foods11182815 (PMC9497606; doi:10.3390/foods11182815)
Supplement: Supplementary file 1 [file foods-11-02815-s001.zip › foods-1842561-supplementary.pdf]

Figure S1. Aroma compositions in four grades PCT obtained from GC–IMS analysis.

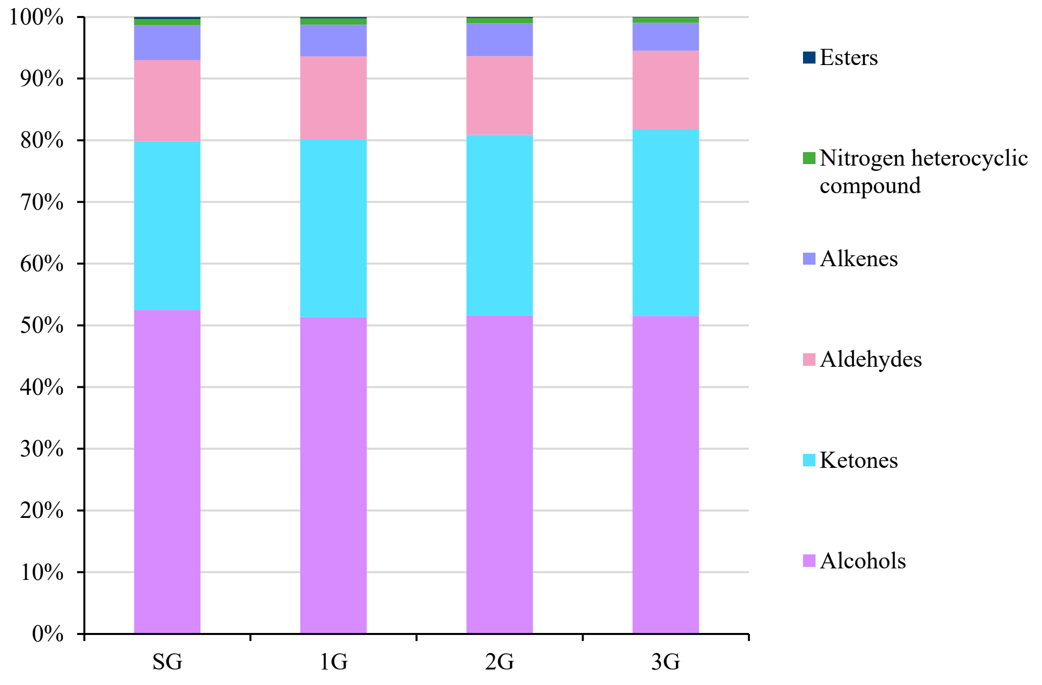

Table S1 Contents of biochemical components in standard samples of different grades of PCT

Classification	Content			
	SG	1G	2G	3G
Gallic acid（mg/g）	1.881±0.122	1.755±0.093	1.581±0.274	1.463±0.028
Catechin（mg/g）	2.435±0.051	1.711±0.086	1.958±0.091	1.572±0.056
Epicatechin（mg/g）	1.251±0.016	1.224±0.466	1.947±0.033	2.112±0.338
Epicatechin gallate（mg/g）	4.890±0.341	4.581±0.223	7.234±0.321	7.173±0.205
Epigallocatechin gallate（mg/g）	81.600±2.048	81.110±1.126	95.830±0.862	95.320±0.555
Epigallocatechin（mg/g）	20.662±0.631	20.031±0.264	17.265±0.221	16.372±0.687
Total Catechin	110.836±0.633	109.130±0.431	124.211±0.380	122.522±0.363
Caffeine（mg/g）	30.201±1.591	28.464±0.375	29.115±0.326	31.266±2.251

Table S2 GC–IMS integration parameters of volatile compounds to distinguish different grades of Tanyang Congou

Count	Compound	CAS#	Formula	MW	RI	Rt [sec]	Dt [a.u.]
1	Octen-3-ol	3391-86-4	C8H16O	128.2	997.3	574.633	1.175
2	α-Terpineol	98-55-5	C10H18O	154.3	1160.8	1030.516	1.199
2'	α-Terpineol (dimer)	98-55-5	C10H18O	154.3	1160.3	1028.607	1.294
3	Linalool	78-70-6	C10H18O	154.3	1101.6	833.327	1.218
3'	Linalool(dimer)	78-70-6	C10H18O	154.3	1088.8	795.828	1.747
4	1,8-Cineole	470-82-6	C10H18O	154.3	1040.6	669.972	1.741
5	1-Octen-3-ol	3391-86-4	C8H16O	128.2	990.4	560.749	1.158
5'	1-Octen-3-ol(dimer)	3391-86-4	C8H16O	128.2	989.4	558.907	1.593
6	2-Hexen-1-ol	2305-21-7	C6H12O	100.2	844	337.616	1.173
7	2-Phenylethanol	1960/12/8	C8H10O	122.2	1111	861.890	1.293
7'	2-Phenylethanol(dimer)	1960/12/8	C8H10O	122.2	1106.6	848.481	1.519
8	n-Hexanol	66-25-1	C6H12O	100.2	764.7	258.826	1.260
9	1-Octanol	111-87-5	C8H18O	130.2	1053.1	700.615	1.457
10	1-Pentanol	71-41-0	C5H12O	88.1	747.2	244.437	1.261
11	1-Butanol	71-36-3	C4H10O	74.1	636.7	172.019	1.196
12	1-Propanol	71-23-8	C3H8O	60.1	524.9	123.653	1.118
13	Propyl hexanoate	626-77-7	C9H18O2	158.2	1105.1	843.898	1.392
14	5-nonanone	502-56-7	C9H18O	142.2	1085.1	785.402	1.815
15	Furaneol	3658-77-3	C6H8O3	128.1	1055.3	706.008	1.619
16	Acetophenone	98-86-2	C8H8O	120.2	1024.1	631.821	1.192
17	2-Heptanone	110-43-0	C7H14O	114.2	886.7	390.617	1.629
17'	2-Heptanone(dimer)	110-43-0	C7H14O	114.2	887.1	391.171	1.261
18	Cyclohexanone	108-94-1	C6H10O	98.1	889.1	393.944	1.462
19	Hexan-2-one	591-78-6	C6H12O	100.2	809.2	300.153	1.492
20	2-Hexanone	591-78-6	C6H12O	100.2	786.8	278.528	1.505
21	2-Butanone	78-93-3	C4H8O	72.1	589.5	149.126	1.244
22	2,3-Butanedione	431-03-8	C4H6O2	86.1	552.2	133.683	1.156
23	Benzaldehyde	100-52-7	C7H6O	106.1	969.1	520.198	1.148
23'	Benzaldehyde(dimer)	100-52-7	C7H6O	106.1	987.5	555.154	1.463
24	Octanal	124-13-0	C8H16O	128.2	983.2	546.768	1.399
25	Heptanal	111-71-7	C7H14O	114.2	898.8	407.252	1.339
25'	Heptanal(dimer)	111-71-7	C7H14O	114.2	898.8	407.252	1.695
26	Pentanal	110-62-3	C5H10O	86.1	737.5	236.800	1.431
27	Butanal	123-72-8	C4H8O	72.1	555.6	135.006	1.278
28	Propanal	123-38-6	C3H6O	58.1	469.5	106.167	1.046
29	Hexanal	111-27-3	C6H14O	102.2	869.6	368.384	1.329
30	Limonene	138-86-3	C10H16	136.2	1012.8	607.012	1.222
31	α-Pinene	80-56-8	C10H16	136.2	919.6	437.648	1.301
32	Styrene	100-42-5	C8H8	104.2	911	424.772	1.441
33	(Z)-3-Hexen-1-ol	928-96-1	C6H12O	100.2	855.2	350.693	1.521
33'	(Z)-3-Hexen-1-ol(dimer)	928-96-1	C6H12O	100.2	855.2	350.693	1.239

34	2,5-Dimethylpyrazine	123-32-0	C6H8N2	108.1	911.4	425.395	1.119

Rt: Represented the retention time in the capillary GC column.

RI: Represented the retention index calculated using n-alkanes C9–C27 as external standard on FS-SE-54-CB-1 column.

Dt: Represented the drift time in the drift tube.

Table S3 The VIP value

Var ID (Primary)	M2.VIPpred
EGC	1.444
Polyphenols	1.343
Heptanal	1.307
GA	1.290
C	1.237
CAF	1.197
1-Pentanol	1.180
Propyl hexanoate	1.173
Acetophenone	1.168
Linalool	1.166
TR/TF	1.156
2-Hexanone	1.151
5-Nonanone	1.144
Cyclohexanone	1.127
Hexanal	1.117
Free amino acids	1.111
Pentanal	1.099
2-Heptanone	1.082
EC	1.073
2,5-Dimethylpyrazine	1.019
Styrene	1.017
Heptanal (dimer)	1.009
2-Hexen-1-ol	1.004
Octanal (dimer)	1.000

Table S4 Pearson correlation coefficient between aroma and VOCs

	Floral	Caramel	Grassy	Fresh	Fruity
Heptanal	-0.94	-0.98	0.91	-0.77	-0.84
1-Pentanol	0.72	0.80	-0.99	0.95	0.82
Propyl hexanoate	0.74	0.86	-1.00	0.90	0.88
Acetophenone	-0.88	-0.82	0.96	-0.97	-0.72
Linalool	0.36	0.69	-0.84	0.66	0.91
2-Hexanone	-0.73	-0.97	0.93	-0.69	-0.98
5-Nonanone	-0.49	0.03	-0.03	-0.24	0.41
Cyclohexanone	0.90	0.97	-0.96	0.82	0.87
Hexanal	0.94	0.94	-0.95	0.85	0.81
Pentanal	-0.94	-0.78	0.88	-0.93	-0.59
2-Heptanone	-0.97	-0.96	0.86	-0.72	-0.77
2,5-Dimethylpyrazine	0.95	0.96	-0.93	0.82	0.82
Styrene	-0.92	-0.79	0.54	-0.42	-0.47
Heptanal (dimer)	-0.90	-0.92	0.98	-0.90	-0.83
2-Hexen-1-ol	-0.89	-0.84	0.96	-0.96	-0.73
Octanal (dimer)	-0.78	-0.40	0.56	-0.78	-0.11

Table S5 Pearson correlation coefficient between taste and biochemical composition

	Mellow	Sweet aftertaste	Astringent	Sour	Bitter
EGC	0.88	0.93	-0.97	-0.98	-0.93
Polyphenols	0.97	0.91	-0.77	-0.77	-0.63
GA	0.96	0.98	-0.95	-0.96	-0.86
C	0.92	0.83	-0.62	-0.60	-0.33
CAF	-0.21	-0.18	0.22	0.29	0.66
TR/TF	0.98	0.99	-0.92	-0.90	-0.65
EC	-0.80	-0.87	0.95	0.97	0.98
Free amino acids	0.94	0.97	-0.96	-0.97	-0.88
